# Concurrence of osteonecrosis and steroid myopathy secondary to oral steroid therapy in a patient with ABCB1 gene polymorphisms: A case report

**DOI:** 10.3389/fendo.2022.1016687

**Published:** 2022-10-07

**Authors:** Yidan Hu, Chenyang Lu, Hui Lin

**Affiliations:** Department of Rheumatology and Immunology, West China Hospital, Sichuan University, Chengdu, China

**Keywords:** steroids, osteonecrosis, steroid myopathy, genotyping, adverse event

## Abstract

Glucocorticoids (GCs) are widely used in various autoimmune diseases. Side effects may occur in patients with long-term or high-dose GC usage. Among them, steroid myopathy and osteonecrosis are two severe forms. We report a patient with pemphigus vulgaris on GC-treatment who developed muscle weakness when a cumulative dose of methylprednisolone reached about 20g (14-80mg/d for 2.5 years). Laboratory tests showed slightly elevated lactate dehydrogenase and hydroxybutyrate dehydrogenase. MRI revealed osteonecrosis in the femoral head, distal femur, and proximal tibia of both legs. The biopsy of the right quadriceps revealed atrophy of type II myofiber without leukocyte infiltration, which was suggestive of steroid myopathy. Genotyping of the patient showed 5G/5G genotype of the PAI-1 gene and CC genotype of the ABCB1 gene (C3435T), suggesting she was sensitive to GCs. The patient’s lesions were considered to be GC-induced adverse events, which were improved with tapering GC. Therefore, it is important to recognize steroid-induced musculoskeletal side effects and genotyping favors personalized medication.

## Introduction

Glucocorticoid (GCs) are a class of steroid hormones that have been widely used clinically as anti-inflammatory, immunosuppressive, and antishock drugs. However, the usage of GCs is limited by various side effects that may involve most major systems including musculoskeletal, cardiovascular, gastrointestinal, endocrine, immunologic, and neuropsychiatric systems (1). Osteonecrosis, also known as ischemic necrosis or avascular necrosis, is a serious condition involving bone destruction, which can lead to progressive joint pain and loss of function. Apart from trauma, the use of corticosteroids is the most common cause of osteonecrosis (2). The risk of osteonecrosis usually increases with high doses and prolonged administration of GCs (2, 3). Muscle involvement is easily under-recognized due to its unspecific symptoms which are often covered by the patient’s underlying disease (4, 5). As the most common type of drug-induced myopathy, steroid myopathy is featured by fatigue, painless muscle weakness, and atrophy (5). The underlying mechanisms for GC-associated side effects are complex. Certain side effects, including diabetes and glaucoma, are predominantly mediated *via* transactivation, ie., binding of the activated GC receptor-DNA to GC-response elements in the promoter or enhancer region of responsive genes, followed by enhanced gene transcription such as gluconeogenic enzymes (3). Whereas, other conditions such as suppression of hypothalamic-pituitary-adrenal axis and GC-associated skin atrophy are predominantly mediated *via* transrepression. The suppression in gene transcription could due to interference with the binding of essential transcription factors or due to that GC receptor may interact with other transcription factors such as Smad 3, nuclear factor-κB and activator protein-1, preventing an activation of gene transcription by these factors. However, in some adverse events such as osteoporosis and steroid myopathy, both transactivation and transrepression are involved (3). Genetic polymorphisms are reported to be associated with risk of GC-induced osteonecrosis (6). However, little is known about the risk factors for steroid myopathy, and the concurrence of osteonecrosis and myopathy secondary to GC treatment is rarely reported. Here we report a patient who developed osteonecrosis and steroid myopathy with the treatment of GCs, and genotyping found ABCB1 gene polymorphisms may account for those observed side effects of GC.

## Case presentation

**Figure 1:**
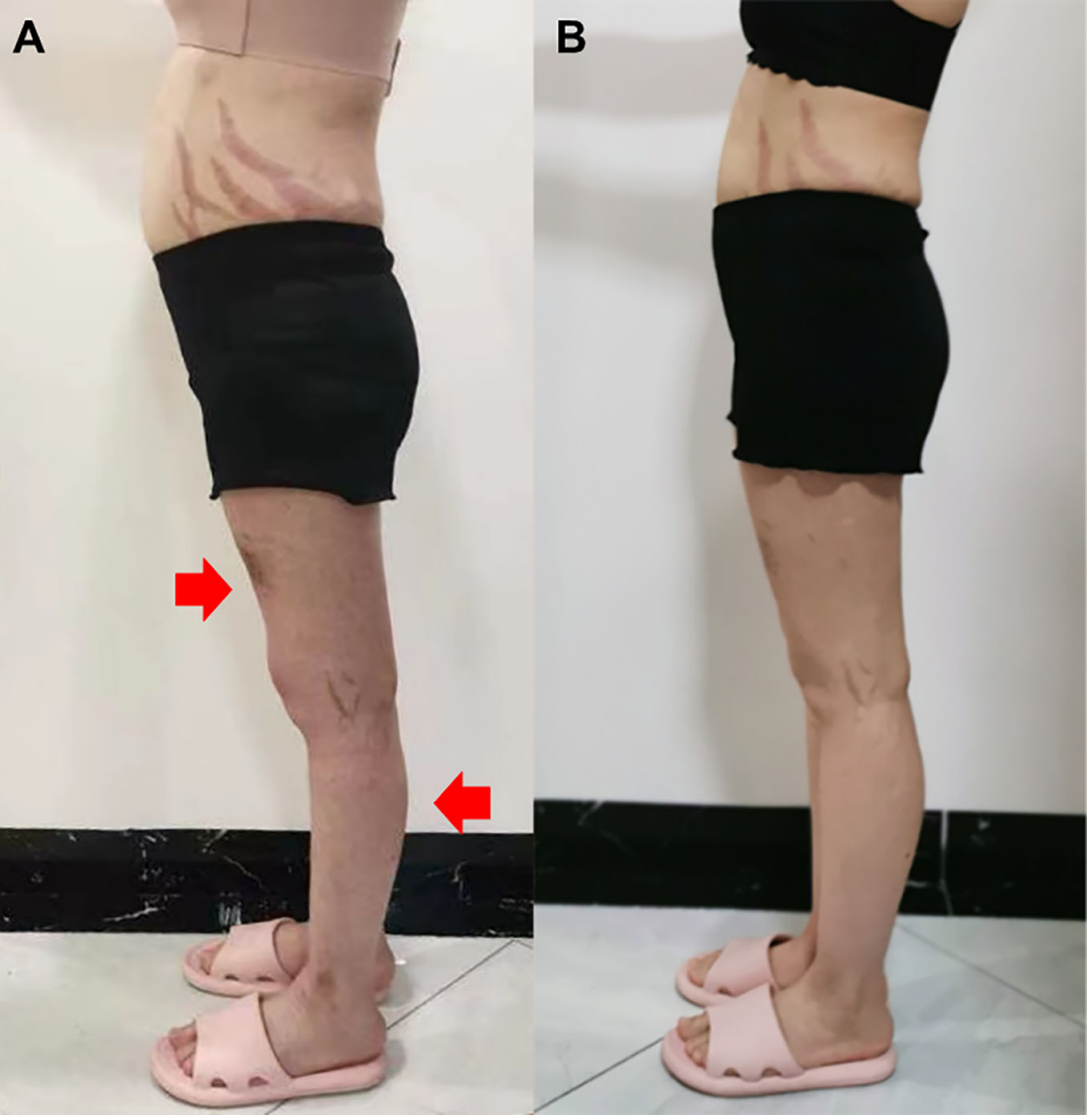
Side view of the patient before glucocorticoid decrement **(A)** and 1 year after glucocorticoid decrement **(B)**. Arrows indicate sites with apparent muscle atrophy.

**Figure 2:**
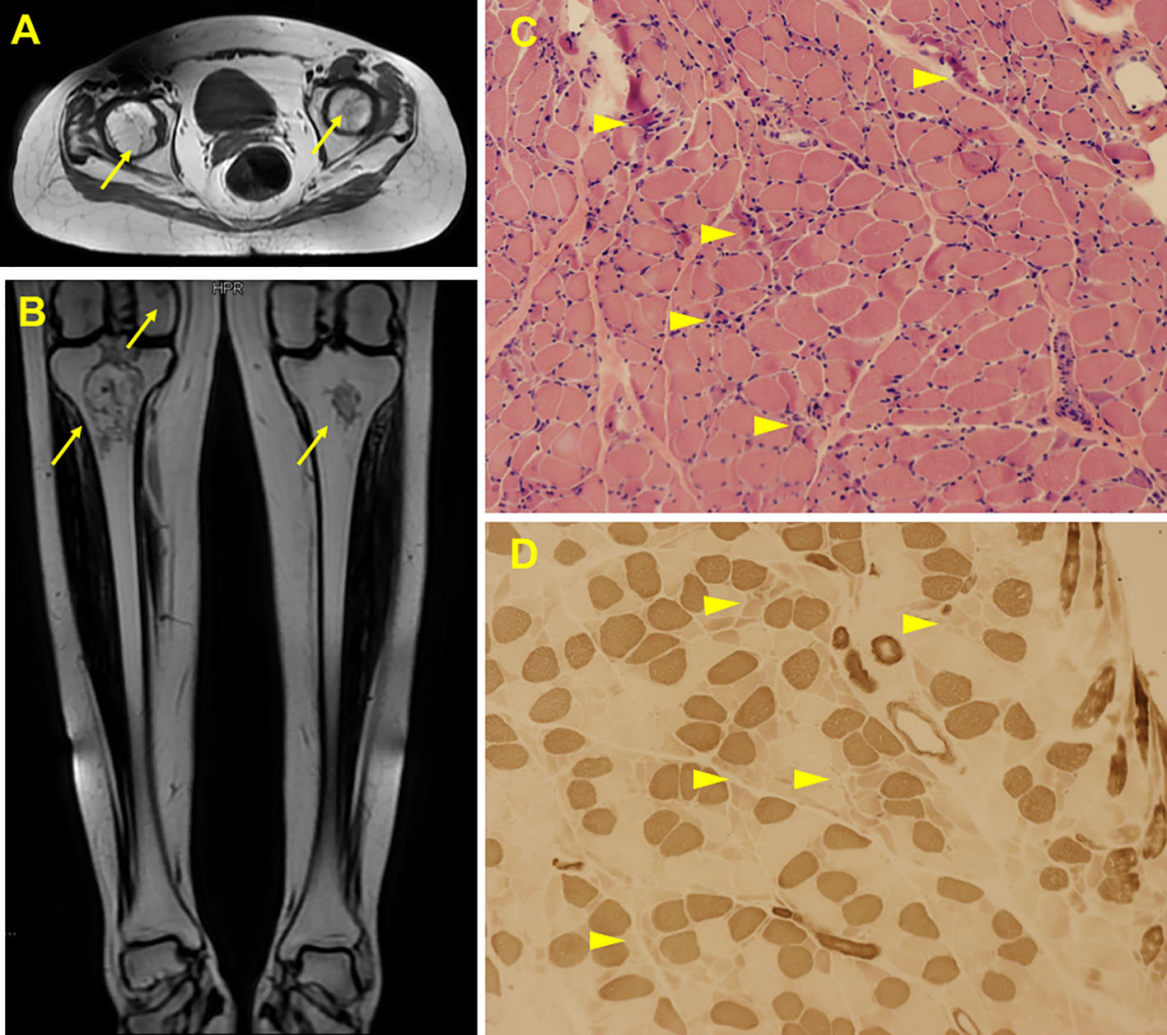
Radiological and histological findings of the case. MRI scan showed abnormal signals in bilateral femoral head **(A)**, distal femur and upper tibia **(B)**, suggesting ischemic necrosis or infarction. Arrows indicate the lesions. H&E staining of the biopsy showed scattered myofiber atrophy **(C)**. ATPase staining revealed atrophy of type II myofibers **(D)**. Arrow heads indicate the lesions.

A 22-year-old female presented with a 5-month history of muscle weakness in both arms and legs. The patient had a history of pemphigus vulgaris for 2 years and received oral methylprednisolone (80 mg/d) and cyclophosphamide (CTX, 50mg/d) for 3 days in the hospital and then was discharged. She had 43mg/d methylprednisolone (tapered down to 14mg/d) combined with 50mg/d CTX for 25 months. During the treatment, she experienced recovery and recurrence of the skin lesions and the dose of methylprednisolone was adjusted accordingly. 5 months before presentation, she developed fungal infection in the skin and diabetes. CTX was discontinued and itraconazole was administered to control the fungal infection, and insulin was injected subcutaneously to overcome blood glucose rises. The cumulative dose of methylprednisolone reached about 20g over the 2.5 years. Physical examination revealed moon face, violaceous striae on the skin of proximal limbs and abdomen, and muscular dystrophy of both legs (**Figure 1A**). No blister, crust, or abscess was found on the skin. Decreased myodynamia was found in lower proximal limbs (Grade 4, MRC scale), but was absent in the upper limbs and lower distal limbs (Grade 5, MRC scale). Laboratory tests showed slightly elevated lactate dehydrogenase (LDH) and hydroxybutyrate dehydrogenase (HBDH), but normal aspartate transaminase (AST) and creatine kinase (CK). The blood glucose was normal, while the serum glycosylated hemoglobin was elevated. Complements, C-reactive protein, and thyroid hormones were within the normal range. Antinuclear antibodies, extractable nuclear antigen antibodies, anti-phospholipid antibodies, and anti-neutrophil cytoplasmic antibodies were negative. MRI revealed osteonecrosis in the femoral head, distal femur, and proximal tibia of both legs (**Figures 2A, B**). The needle electromyography showed shortened duration of the motor unit action potential in the right deltoid muscle, left biceps branchii, and right gastrocnemius, and low amplitude in the right deltoid muscle and left biceps branchii with mixed firing pattern and interference pattern. No denervation potential was detected in these muscles. Myogenic damage was considered and a muscle biopsy of the right quadriceps was performed. Histological examinations revealed atrophy of type II myofiber without significant leukocyte infiltration, which were suggestive of steroid myopathy (**Figures 2C, D**). The cooccurrence of these two severe steroid-associated adverse events made us consider she may be sensitive to GCs and prompted us to check the presence of susceptible genotypes to steroids in this patient. Genotyping showed C/C allele of the ABCB1 gene (C3435T) and 5G/5G allele of the PAI-1 gene. The osteonecrosis and myopathy in this patient were considered to be secondary to GC-administration. Given the pemphigus vulgaris was alleviated, no additional treatment was given. The dose of methylprednisolone was tapered to 4mg every other day to avoid skin lesion relapses and her muscle weakness attenuated. The patient also received calcium supplement (600mg/d) and calcitriol (0.25μg/d) to slow the progression of osteonecrosis. During a 1 year of follow-up, her muscle strength recovered and muscular dystrophy also improved (**Figure 1B**).

## Discussion

Osteonecrosis and myopathy are common complications of GC administration in terms of bone and muscle, respectively. However, the concurrence of them is rarely reported possibly due to overlook of either condition. Drigo I et al. (7) described a child with increased number of GC receptors developed osteonecrosis and recurrent myopathy with steroid therapy. Here we reported concurrence of GC-induced osteonecrosis and pathologically confirmed myopathy in a patient with ABCB1 polymorphisms.

GC-induced osteonecrosis is the most common cause of nontraumatic osteonecrosis with a prevalence of 3%-38% (8). The femoral head is the most commonly affected area, followed by knee, humeral head, and ankle (2). Multiple factors are involved in the development of osteonecrosis, including thrombophilia, endothelial cell damage, activation of coagulation pathway, and lipid metabolism dysregulation (9, 10). Systemic GC at a high cumulative dose or high peak dose in a short time is a risk factor for GC-associated osteonecrosis (2). Majority of osteonecrosis cases involved high dose pulse steroids with dexamethasone or methylprednisolone, followed by varying durations of oral steroids on a daily or repetitive basis. The reported cumulative dose ranged from 150mg to 445000mg of prednisone equivalent (average 5969mg prednisone). Approximately 1000mg of oral prednisone administered within a short window will place patients at increased risk. Any addition of parenterally administered steroids, further increases this risk significantly (2). Among patients with systemic lupus erythematosus, nearly all patients who developed osteonecrosis had average >20mg/d prednisone or >40mg/d in the highest month of therapy (11). There is also a positive correlation with osteonecrosis when patients were treated with a cumulative dose of >12g/year (12). A meta-analysis pooled data from 1137 recovered severe acute respiratory syndrome patients to determine the incidence of osteonecrosis with different doses of steroids (13). They found a non-linear relationship between rate ratio of osteonecrosis and steroid doses, which indicated the risk of osteonecrosis increased from cumulative 5g of methylprednisolone and peaked between 10 and 15g. A cumulative dose of methylprednisolone ≤5 g and duration ≤30 days resulted in a relatively low risk of osteonecrosis (13). Till now, no efficient therapy is available for osteonecrosis, and joint replacement may be inevitable if the disease progresses and non-operative treatments failed.

GC-induced myopathy is a highly prevalent toxic noninflammatory myopathy, which is characterized by fast-twitch or type II muscle fiber atrophy (5, 14). Dexamethasone could induce selective loss of myosin heavy chain protein, breaking down the muscle integrity (15). Furthermore, myosin filament degradation in steroid myopathy is similar to that in critical illness myopathy, a type of myopathy usually develops in severely ill patients. It is believed that steroids may play an important role in muscle wasting in critical illness myopathy, since a high cortisol level is a feature of the stress response in patients with multiple organ failure (16). In addition, muscle denervation and concomitant high-dose dexamethasone led to severe muscle atrophy and selective myosin heavy chain depletion in rodents, which is identical to pathological findings in critical illness myopathy (17).

Steroid myopathy usually has an indolent presentation and predominantly affects pelvic girdle muscles. Muscle weakness and atrophy are common, while pain is often absent (5). Steroid myopathy is largely overlooked by physicians for lack of specific symptoms and biomarkers. It should be taken into consideration if patients developed proximal muscle weakness, fatigue, reduced muscle performance with history of steroids usage (4). The pathophysiology of steroid-induced myopathy is multifactorial. It may be related to the influences of GCs on muscle cell metabolism and functional activities by inhibiting protein synthesis and destroying mitochondrial structure (14). Corticosteroid-induced myopathy typically develops with doses >10mg/d of prednisone equivalents used for four weeks or longer. Two to three weeks of higher doses (e.g., 40-60 mg prednisone/day) have been associated with more acute presentations (18). Steroid myopathy may be reversible with reduction or discontinuation of steroids, though this may take several months to a year (4, 18). In this case, the patient had attenuated muscle presentations with tapering GC, confirming the diagnosis of steroid myopathy.

CTX is a very effective treatment for many autoimmune diseases including pemphigus vulgaris, decreasing the cumulative dose of systemic GC required (19). There is a concern that CTX may be associated with bone avascular necrosis for it causes endothelial dysfunction (20) and influences bone mineralization (21). However, osteonecrosis caused by CTX monotherapy is uncommon and usually happens in complicated conditions. It is reported that CTX could increase the risk of bisphosphonate-related osteonecrosis of the jaw (BRONJ) among multiple myeloma patients who are receiving pamidronate, a bisphosphonate (22). In mice, high dose CTX is associated with the development of BRONJ following tooth extraction only when CTX is used together with bisphosphonate (23). Animal toxicological experiments on rodents revealed that CTX administration could lead to persistent muscle impairment (24). However, CTX is used as a treatment escalation when standard immunosuppression and intravenous immunoglobulin are insufficient in severe myositis or with vital organ involvement (25). There is only one case reporting acute rhabdomyolysis following administration of high-dose CTX (26). Our patient only had low dose of CTX for a short period, therefore, we speculate CTX may not be the main cause of osteonecrosis and myopathy in this case.

GCs induced generation of reactive oxygen species and strongly up-regulated the stress-response transcription factors including FOXO1 and FOXO3A (27). The elevated FOXOs may be involved in GC-induced osteonecrosis as FOXOs is able to attenuate bone formation by suppressing Wnt signaling (28). GC-induced muscle atrophy is mediated by FOXOs as FOXO1 and the glucocorticoid receptor synergistically activate the MuRF1 gene which is associated with skeletal muscle atrophy (29). Interestingly, FOXO1 and FOXO4 subtype are positive regulators of ABCB1 (30) and PAI-1 (31), respectively.

Genetic variants of PAI-1 gene and ABCB1 gene are associated with steroid-induced femoral head osteonecrosis (32). Plasminogen activator inhibitor type 1 (PAI-1) is a glycoprotein regulating fibrinolysis by forming complexes with tissue plasminogen activator. High-dose GC could increase plasma PAI-1 level, resulting in a relative hypercoagulable status (33). 4G allele of PAI-1 was associated with an increased risk of osteonecrosis compared with the 5G allele (6, 32). Moreover, excessive PAI-1 also result in increased muscle inflammation and impaired regeneration of muscle, favoring muscle atrophy and fibrosis (34).

The ABCB1 gene encodes a transport protein, p-glycoprotein (P-gp), which has crucial roles in pumping foreign substances out of cells. Enhanced P-gp function was able to decrease the risk of steroid-induced osteonecrosis through inhibiting adipogenesis and apoptosis of mesenchymal stem/stromal cells (35). Compared to TT and TC genotypes of ABCB1 (C3435T), CC genotype was associated with increased risk of osteonecrosis (6). Moreover, ABCB1 polymorphisms, which lead to P-gp expression reduction or dysfunctional P-gp are also associated with drug-induced myopathy (36, 37). However, ABCB1 activity is not related to GC-susceptibility in murine B subsets (38). Further study of the c.3435 polymorphism or other genetic factors in a large series of steroid myopathy patients is warranted. Our patient, who is highly sensitive to GCs, has 5G/5G genotype of PAI-1 and CC genotype of ABCB1, indicating the importance of ABCB1 and PAI-1 in GC-related side effects are not equal.

Genotyping sometimes could benefit patients on special treatments, especially when the treatments are known to cause severe side effects in susceptible individuals. For example, genotyping of thiopurine methyltransferase (TPMT) and nudix hydrolase 15 (NUDT15) were recommended for patients who experience severe bone marrow toxicities or repeated episodes of myelosuppression before azathioprine administration, for azathioprine is metabolized by TPMT and NUDT15 or could lead to severe myelosuppression if patients have defects in those two enzymes (39). Genotyping steroid side effect-risk genes are recommended for patients who have steroid-induced adverse events frequently or experience severe forms, and steroids dose reduction or alternative therapy should be considered. Compared to the financial burdens of joint replacement due to steroid-induced osteonecrosis or labor service due to muscle dysfunction, prompt genotyping is economic to some extent. However, which gene is appropriate for screening and is this strategy cost-effective warrant further study.

In summary, we reported a unique case of osteonecrosis and myopathy secondary to GC usage with ABCB1 CC genotype. Genotyping of steroid side effects-risk genes are recommended to be performed on patients who repeatedly experienced GC-associated adverse events or had severe forms, favoring personalized medication.

## Data availability statement

The original contributions presented in the study are included in the article. Further inquiries can be directed to the corresponding author.

## Ethics statement

The studies involving human participants were reviewed and approved by Ethics Committee of Sichuan University West China Hospital. The patients/participants provided their written informed consent to participate in this study. Written informed consent was obtained from the individual(s) for the publication of any potentially identifiable images or data included in this article.

## Author contributions

YH and HL had full access to all the data in the study and takes responsibility for the integrity of the data. YH and HL took care of the patient and collected the clinical information. CL did the literature review and drafted the manuscript. All authors were involved in drafting the manuscript or revising it critically. All authors contributed to the article and approved the submitted version.

## Funding

This work was supported by the National Natural Science Foundation of China (No. 81102274) and the Sichuan Science and Technology Program (2017HH0110).

## Conflict of interest

The authors declare that the research was conducted in the absence of any commercial or financial relationships that could be construed as a potential conflict of interest.

## Publisher’s note

All claims expressed in this article are solely those of the authors and do not necessarily represent those of their affiliated organizations, or those of the publisher, the editors and the reviewers. Any product that may be evaluated in this article, or claim that may be made by its manufacturer, is not guaranteed or endorsed by the publisher.
